# Electrospun naringin-loaded microsphere/sucrose acetate isobutyrate system promotes macrophage polarization toward M2 and facilitates osteoporotic bone defect repair

**DOI:** 10.1093/rb/rbad006

**Published:** 2023-02-20

**Authors:** Jihong Li, Jinlin Song, Di Meng, Yin Yi, Ting Zhang, Yu Shu, Xiaohong Wu

**Affiliations:** Stomatological Hospital of Chongqing Medical University, No. 426 Songshibei Road, Yubei District, Chongqing 401147, China; Chongqing Key Laboratory of Oral Diseases and Biomedical Sciences, Chongqing 401147, China; Chongqing Municipal Key Laboratory of Oral Biomedical Engineering of Higher Education, Chongqing 401147, China; Stomatological Hospital of Chongqing Medical University, No. 426 Songshibei Road, Yubei District, Chongqing 401147, China; Chongqing Key Laboratory of Oral Diseases and Biomedical Sciences, Chongqing 401147, China; Chongqing Municipal Key Laboratory of Oral Biomedical Engineering of Higher Education, Chongqing 401147, China; Stomatological Hospital of Chongqing Medical University, No. 426 Songshibei Road, Yubei District, Chongqing 401147, China; Chongqing Key Laboratory of Oral Diseases and Biomedical Sciences, Chongqing 401147, China; Chongqing Municipal Key Laboratory of Oral Biomedical Engineering of Higher Education, Chongqing 401147, China; Stomatological Hospital of Chongqing Medical University, No. 426 Songshibei Road, Yubei District, Chongqing 401147, China; Chongqing Key Laboratory of Oral Diseases and Biomedical Sciences, Chongqing 401147, China; Chongqing Municipal Key Laboratory of Oral Biomedical Engineering of Higher Education, Chongqing 401147, China; Stomatological Hospital of Chongqing Medical University, No. 426 Songshibei Road, Yubei District, Chongqing 401147, China; Chongqing Key Laboratory of Oral Diseases and Biomedical Sciences, Chongqing 401147, China; Chongqing Municipal Key Laboratory of Oral Biomedical Engineering of Higher Education, Chongqing 401147, China; Stomatological Hospital of Chongqing Medical University, No. 426 Songshibei Road, Yubei District, Chongqing 401147, China; Chongqing Key Laboratory of Oral Diseases and Biomedical Sciences, Chongqing 401147, China; Chongqing Municipal Key Laboratory of Oral Biomedical Engineering of Higher Education, Chongqing 401147, China; Stomatological Hospital of Chongqing Medical University, No. 426 Songshibei Road, Yubei District, Chongqing 401147, China; Chongqing Key Laboratory of Oral Diseases and Biomedical Sciences, Chongqing 401147, China; Chongqing Municipal Key Laboratory of Oral Biomedical Engineering of Higher Education, Chongqing 401147, China

**Keywords:** electrospun microspheres, macrophage polarization, Ng-m-SAIB, osteoporotic bone defects

## Abstract

Repairing osteoporotic bone defects is still a major clinical challenge. Recent studies have revealed that immune response is also essential in osteogenesis. The intrinsic inflammatory response of the host, especially the M1/M2 polarization status and inflammatory secretory function of macrophages, can directly affect osteogenic differentiation. Therefore, in this study, an electrospun naringin-loaded microspheres/sucrose acetate isobutyrate (Ng-m-SAIB) system was constructed to investigate its effect on the polarization of macrophage and osteoporotic bone defects. The results of both *in vitro* and *in vivo* experiments showed that Ng-m-SAIB had good biocompatibility and could promote the polarization of macrophage toward M2, thereby forming a favorable microenvironment for osteogenesis. The animal experiments also showed that Ng-m-SAIB could promote the osteogenesis of critical size defects in the skull of the osteoporotic model mouse (the senescence-accelerated mouse-strain P6). Together, these results collectively suggested that Ng-m-SAIB might be a promising biomaterial to treat osteoporotic bone defects with favorable osteo-immunomodulatory effects.

## Graphical Abstract

**Figure:**
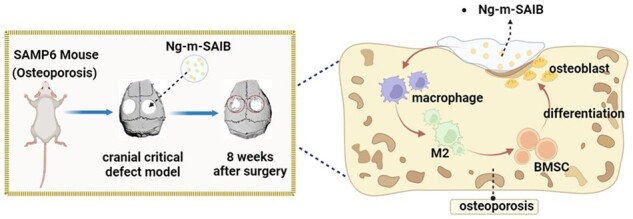

## Introduction

With the aging of society, osteoporosis has become a major health issue, and the treatment of osteoporotic bone defects faces great challenges [1, 2]. The current conventional treatment methods for osteoporotic bone defects include oral medications, bone grafting and alternative materials for bone tissue engineering; however, all of them have numerous limitations [3, 4]. Among them, oral drugs have a low absorption rate, insignificant targeting effect, gastrointestinal tolerance and other adverse effects [5]. Allogeneic bone grafting suffers from blood-borne contamination and rejection during the grafting process; therefore, it is now used less frequently. Autologous bone grafting is the gold standard for the treatment of conventional bone defects. However, the autologous bones of osteoporotic patients are in a pathological state and might not play the osteoinductive filling role after implantation at the bone defect site [6, 7]. These limitations in conventional therapies have urged researchers to develop more effective and safer tissue engineering strategies for the treatment of osteoporotic bone defects [8, 9].

Naringin, a flavonoid, is an important active ingredient in the traditional Chinese medicine’s bone tonic [10]. It can promote osteogenesis and inhibit osteolysis, showing effectiveness against osteoporosis [11, 12]. However, studies have shown the poor oral utilization of naringin; therefore, selecting a suitable drug carrier can optimize the delivery, action and persistence of naringin at the target site [13]. Electrospun microsphere, having excellent potential for drug delivery and sustained release of encapsulated material, has the advantages of low production cost, good reproducibility and high drug encapsulation rate [14, 15]. Studies have also shown that mixing the microspheres with sucrose acetate isobutyrate (SAIB) results in the formation of a better sustained drug delivery system [16]. The injectable nature of the SAIB matrix enables its use in a wider range of minimally invasive medical treatments, such as fractures and periapical lesions; this is in line with the minimally invasive concept encouraged in clinical practices [17, 18]. The sustained-release system also provides more control over the duration and dose of drug release, thereby making it a more cost-effective and safer method of drug administration. This local delivery method has a higher drug absorption rate as compared to systemic oral administration [19]. In our previous studies, the electrospun naringin-loaded microsphere/SAIB system (Ng-m-SAIB) prepared by our research group showed good slow-release performance and significant osteogenic effects on the cranial bone defects in Sprague Dawley rats [20, 21]. However, its potential to treat osteoporotic bone defects has not been investigated yet; moreover, its osteogenic mechanism is also not clear.

Recent studies have shown that immune response is also an important osteogenesis-associated component [22, 23]. Macrophages are the main immune cells, which initiate and maintain the inflammatory response and are directly involved in the osteogenesis process by secreting important osteogenesis-related cytokines [24, 25]. In response to microenvironmental stimulation, macrophages can polarize into M1 and M2 types. Among these types, M1 macrophages promote the development of inflammation, while M2 macrophages can secrete anti-inflammatory factors to promote tissue repair [26, 27]. Many biomaterials are currently being used to promote osteogenesis by inducing the polarization of macrophages into M2 type, and their effects have been widely reported [28–33].

In this study, the Ng-m-SAIB was prepared. It was hypothesized that Ng-m-SAIB could regulate the polarization of macrophages into M2 type and form a microenvironment favorable for osteogenesis, thereby promoting osteogenesis in the osteoporotic bone defects. Therefore, the main objective of this study was to investigate the immunomodulatory functions of Ng-m-SAIB and its osteogenic effects on osteoporotic bone defects.

## Materials and methods

### Preparation of the Ng-m-SAIB and m-SAIB

The Ng-m-SAIB and blank microsphere/SAIB (m-SAIB) were prepared as described in our previous study [21]. The preparation procedure has also been explained in the Supplementary data.

### Cell culture

The RAW264.7 cells (murine monocyte/macrophage cell line) and bone marrow stromal cells (BMSCs) were cultured in a configured high-sugar medium. The components of the cell culture medium were 89% DMEM (Hyclone, USA), 10% fetal bovine serum (Hyclone, USA) and 1% penicillin/streptomycin (Hyclone, USA). Furthermore, the growth medium was replaced every 2–3 days.

### Biocompatibility of Ng-m-SAIB *in vitro*

#### Cell counting kit-8 assay

The m-SAIB and Ng-m-SAIB were spread in the center of wells in a 96-well plate and sterilized under ultra-violet (UV) light for 2 h. Then, the RAW264.7 cells were seeded into the wells at a density of 3000 cells/well. After the co-culture with Ng-m-SAIB and m-SAIB for 1, 3, 5 and 7 days, the proliferation activities of the RAW264.7 cells were identified using a Cell Counting kit-8 (CCK8) assay kit (Dojindo, Japan). The absorbance (optical density) at 450 nm (OD_450_) was then measured using a spectrometer (Thermo Scientific, USA).

#### Flow cytometry assay

The prepared Ng-m-SAIB (contains 10 mg Ng-m) and m-SAIB (contains 10 mg blank-m) samples were spread in the center of a 10-cm culture dish and sterilized under UV light for 2 h. A blank dish was used as a negative control group. Approximately 20 000 RAW264.7 cells were added to each culture dish. After co-culture for 2 days, cells were digested and collected. The collected cells were stained using the Annexin V-FITC/PI Apoptosis Detection Kit (Yeasen, China). Then the fluorescence signals were detected using the flow cytometry (BD, USA) within 1 h after staining was completed, and the FlowJo software was used to analyze the data.

### Effects of Ng-m-SAIB on the polarization of macrophages *in vitro*

The Ng-m-SAIB was spread in the center of 10-cm culture dishes and the material was sterilized under UV light for 2 h. Then the RAW264.7 cells were co-cultured with or without Ng-m-SAIB for 4 days, and then, the RAW264.7 cells were digested and collected.

#### Quantitative real-time polymerase chain reaction

First, the total RNA from the RAW264.7 cells was extracted using the RNA extraction kit (R0032, Beyotime, China). Then a PrimeScript RT reagent kit (RR047A, TaKaRa, Japan) was used to reverse the RNA into cDNA. Quantitative real-time polymerase chain reaction (RT-PCR) was performed with the PrimeScript RT-PCR kit (RR820A, TaKaRa, Japan). The RT-PCR reaction proceeded at 95°C for 30 s, 95°C for 5 s and 60°C for 30 s for 40 cycles. Gene expression was calculated with the 2^–△△^Ct method. The primers used in this study are listed in Table 1.

**Table 1.** Primer sequences for each gene

Genes	Species	Forward (5′–3′)	Reverse (5′–3′)
*GAPDH*	Mouse	CTCCCACTCTTCCACCTTCG	TTGCTGTAGCCGTATTCATT
*CCR7*	Mouse	ACGGGCTGGTGATACTGACG	GCCAGGTTGAGCAGGTAGGT
*CD206*	Mouse	AAAGGCAAGGATGGATACTGG	GCATCAGTGAAGGTGGATAGAGT
*IL10*	Mouse	GACAATAACTGCACCCACTTCC	AGTCGGTTAGCAGTATGTTGTCC
*TNFα*	Mouse	CACCACGCTCTTCTGTCTACTG	GGTCTGGGCCATAGAACTGA

#### Immunofluorescence staining of macrophages

The RAW264.7 cells were co-cultured with Ng-m-SAIB for 4 days and then digested and re-seeded into 24-well plates at a density of 10 000 cells per well, and immunofluorescence staining was performed after the cells were attached to the wells. After the cells were washed three times with Phosphate Buffered Saline (PBS), they were fixed with 4% paraformaldehyde for 20 min and blocked with 5% Bull Serum Albumin for 1 h at room temperature. Then, the cells were incubated with *CD206* antibody (marker of M2 macrophages, 1:200, HUABIO, JF0953) and *CCR7* antibody (marker of M1 macrophages, 1:100, HUABIO, SR36-04), separately, for 1 h. After washing with PBS, the cells were incubated with the respective secondary antibodies (Alexa Fluor 488 or 594 conjugated goat anti-rabbit or anti-mouse IgG; both 1:400; both HUABIO; G210903 and G210923, respectively) for another 1 h. Finally, the DAPI solution was used to stain the cells for 10 min. The staining results were observed and photographed under a fluorescence microscope.

#### Flow cytometry assay

The RAW264.7 cells were co-cultured with Ng-m-SAIB for 4 days and then incubated with the *CD206* antibody (1:100, PE, eBioscience™) or *CD86* antibody (marker of M1 macrophages, 1:200, PE, eBioscience™) at 4°C for 1 h. After 1 h, the excess antibodies were removed by washing with PBS three times. The fluorescence signals were detected using the flow cytometry (BD, USA) within 1 h after staining was completed, and the FlowJo software was used to analyze the data.

### Osteogenic differentiation effect of the conditioned medium

Ng-m-SAIB were placed in a 10-cm cell-culture dish and sterilized under UV light for 4 h before cell seeding. Then, ∼ 300 000 RAW264.7 cells were co-cultured into each culture dish for 4 days. Then, the cell culture medium was collected, and the sediment was removed by centrifugation. The supernatant was then configured with a normal medium in a ratio of 1:2 to form a conditioned medium, which was stored at –80°C until further use.

#### CCK8 assay

The BMSCs were seeded into a 96-well plate, and the density was 1000 per well. On the second day, the experimental group replaced the cell culture medium with a conditioned medium. Then, on Days 1, 3, 5, and 7 of changing the medium, the cell counting was performed using CCK8 assay as described previously in Section ‘Cell counting kit-8 assay’.

#### Alkaline phosphatase staining

The BMSCs were cultured in the normal and conditioned media, respectively. Alkaline phosphatase (ALP) staining was performed on Days 7 and 14. The cells were fixed in 4% paraformaldehyde for 30 min at room temperature, followed by washing with PBS. Then the appropriate amount of the configured staining solution was added according to the instructions of the BCIP/NBT Alkaline Phosphatase Color Development Kit (Beyotime, Shanghai, China). The staining results were observed and photographed under a light microscope (Leica, Germany).

#### Alizarin red staining

The BMSCs were cultured in normal and conditioned media and then stained with alizarin red staining at Day 21. After rinsing three times with PBS, the BMSCs were fixed in 4% paraformaldehyde. Then, appropriate alizarin red staining solution was added (Solarbio, China) to stain the cells for 1 h. The calcium nodules were dissolved by adding 10% cetylpyridinium chloride solution (Sigma, USA) for the semi-quantitative analysis, and the microplate reader (Thermo Scientific, USA) was used to test the optical density at 562 nm.

#### Quantitative real-time polymerase chain reaction

The BMSCs were collected after culturing in two different media for 14 days. Then, follow the method described in Section ‘Quantitative real-time polymerase chain reaction’. The primers used for this experiment are listed in Table 2.

**Table 2.** Primer sequences for each gene

Genes	Species	Forward (5′–3′)	Reverse (5′–3′)
*GAPDH*	Mouse	CTCCCACTCTTCCACCTTCG	TTGCTGTAGCCGTATTCATT
*ALP*	Mouse	TGACTACCACTCGGGTGAACC	TCTGGTGGCATCTCGTTATCC
*RUNX2*	Mouse	TTCCAGACCAGCAGCACTCC	GCTTCCGTCAGCGTCAACAC
*Col1α1*	Mouse	ACGCCATCAAGGTCTACTGC	CGGGAATCCATCGGTCA

### Animal experiments

#### Establishment of the cranial defect model using the senescence-accelerated mouse-strain P6

A total of 24 male senescence-accelerated mouse-strain P6 (SAMP6) mice (16 weeks of age with an average weight of 25–30 g) were purchased from the Department of Laboratory Animal Science, Peking University Health Science Center (Beijing, China) and randomly divided into four groups (*n* = 6), including control, m-SAIB, Ng-SAIB and Ng-m-SAIB groups. All the mice were anesthetized by isoflurane inhalation using a small animal anesthesia machine (RWD, R500, China). After dehairing the top of the mice’s heads, the surgical site was disinfected, and a longitudinal skin incision was made using a scalpel to expose the bone surface. Then, a 3-mm full-thickness bone defect was made by low-speed drilling and saline cooling on both sides of the cranial parietal bone. The bone defects were placed with m-SAIB, Ng-SAIB, Ng-m-SAIB or no material. Their skins were sutured at the end of surgery.

#### Sample harvesting

After 4 and 8 weeks of surgery, the mice in each group were sacrificed using Automated CO^2^ Delivery Systems (LC500, China). The cranial parietal bones of all the mice were removed intact and immersed in 4% paraformaldehyde for fixation. The hearts, livers, spleens, lungs, kidneys and brains of the mice in control and Ng-m-SAIB groups were also collected after 8 weeks.

#### Effects of Ng-m-SAIB on the polarization of macrophages in vivo

First, the skull samples were decalcified by immersing them in a test tube, containing the ethylene diamine tetraacetic acid solution (pH 7.2, Biosharp, China). The test tube was then placed at 37°C in a shaking incubator. The decalcifying solution was refreshed every 2 days to speed up the decalcification process. The skull specimens were paraffinized after decalcification and sectioned. After dewaxing the paraffin sections, the immunofluorescence staining was performed as described in the literature [34]. The following antibodies were used in this study: *F4/80* antibody (macrophage marker, GB113373, 1:300, Servicebio, China), *iNOS* antibody (M1-type macrophage marker, GB11119, 1:300, Servicebio, China), *CD206* antibody (M2-type macrophage marker, GB13438, 1:300, Servicebio, China), *TNFα* antibody (GB23303, 1:2000, Servicebio, China) and *IL10* antibody (GB25303, 1:200, Servicebio, China). After staining, a fluorescence microscope (Nikon, Japan) was used to observe and photograph the sections. The average fluorescence intensity of each sample in the images was analyzed using the ImageJ software.

#### Osteogenic effects of Ng-m-SAIB on osteoporotic bone defects in vivo

The computed tomography (CT) scans of all cranial specimens were performed using the micro-CT (SCANCO, Viva CT40, Switzerland). The 3D image reconstruction was performed using the accompanying SCANCO analysis software, and the bone volume-to-total volume ratio (BV/TV) and bone mineral density (BMD) of the defect sites were identified.

The skull specimens were paraffinized as described in Section ‘Effects of Ng-m-SAIB on the polarization of macrophages *in vivo*’. After dewaxing and hydrating, the paraffinized sections were stained with a hematoxylin–eosin (H&E) staining kit (Solarbio, G1120, China) and a Masson staining kit (Solarbio, G1304, China), following their respective manufacturer’s instructions. The ALP immunofluorescence staining was performed as described previously [34] using the ALP antibody (Servicebio, GB112527, 1:200, China).

#### In vivo biocompatibility of Ng-m-SAIB

The individual organs of the mice in both groups (as described in Section ‘Sample harvesting’) were fixed and paraffinized. The tissue sections were stained with the H&E staining kit (Solarbio, G1120, China) and the tissue morphological changes were observed.

### Statistical analyses

The data were expressed as mean ± standard deviation. All the data analyses were performed using SPSS 20.0 software (IBM, USA). The differences between the groups were analyzed using analysis of variance and Student–Newman–Keuls tests. The significant and highly significant differences were represented by one and two asterisks (*P < 0.05 and **P < 0.01, respectively).

## Results

### Characterization and biocompatibility of Ng-m-SAIB

**Figure 1.**
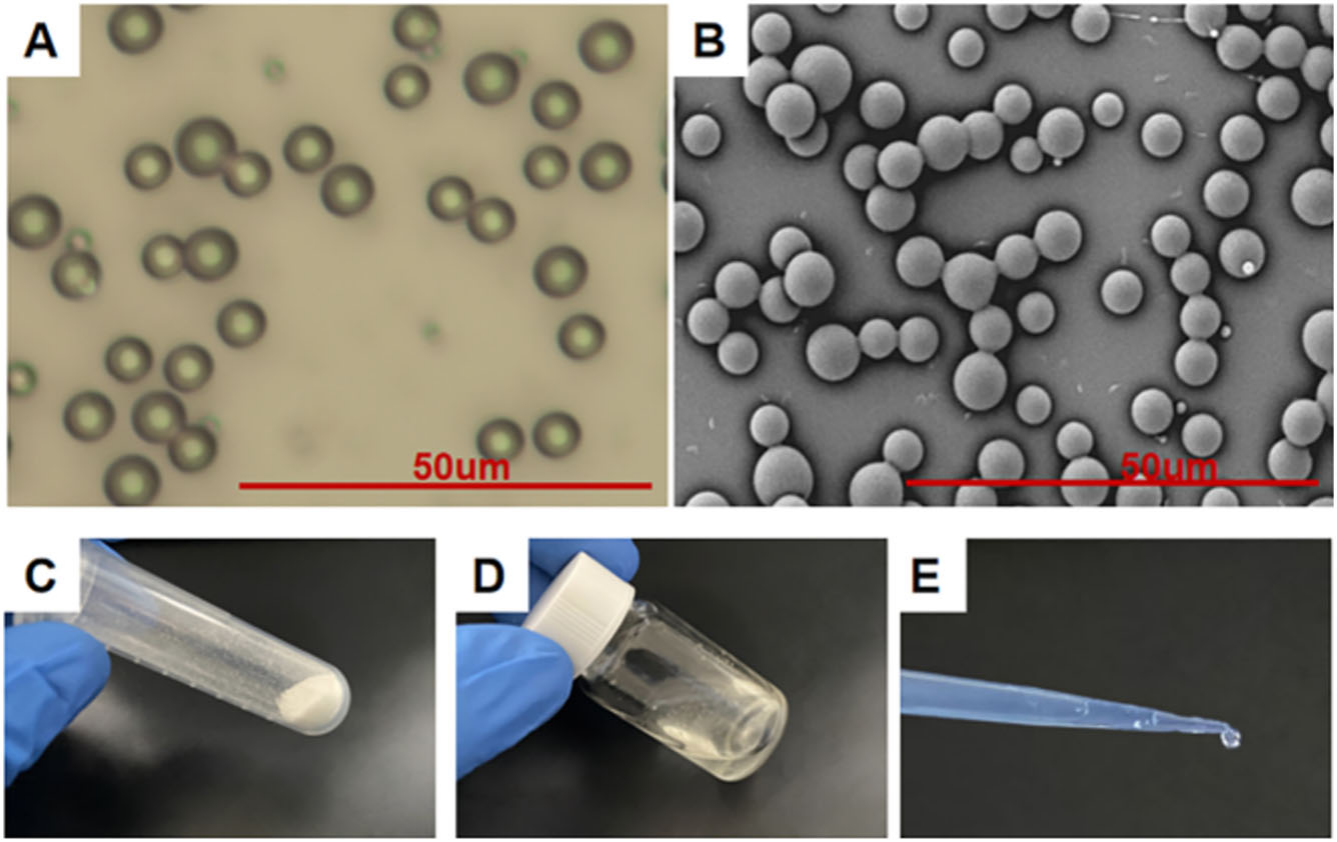
(**A**) Optical image and (**B**) SEM image of Ng-m. Scale bar = 50 μm. (**C**) Ng-m collected in an EP tube. (**D**) Ng-m-SAIB collected in a glass bottle. (**E**) Injectable ability of Ng-m-SAIB.

As shown in Fig. 1A and B, the morphology of Ng-m under the polarized light and scanning electron microscopes showed good monodispersity with smooth surface spheres (5.037 ± 0.172 µm diameter) and good uniformity of particle size distribution. The Ng-m prepared using electrospray technology was dried and collected in an EP tube (Fig. 1C), which appeared as a fine white powder. SAIB was a colorless, transparent and fluid viscous liquid at room temperature. After mixing the Ng-m with SAIB magnetically (Fig. 1D), the Ng-m-SAIB still exhibited good fluidity and injectable properties (Fig. 1E).

**Figure 2.**
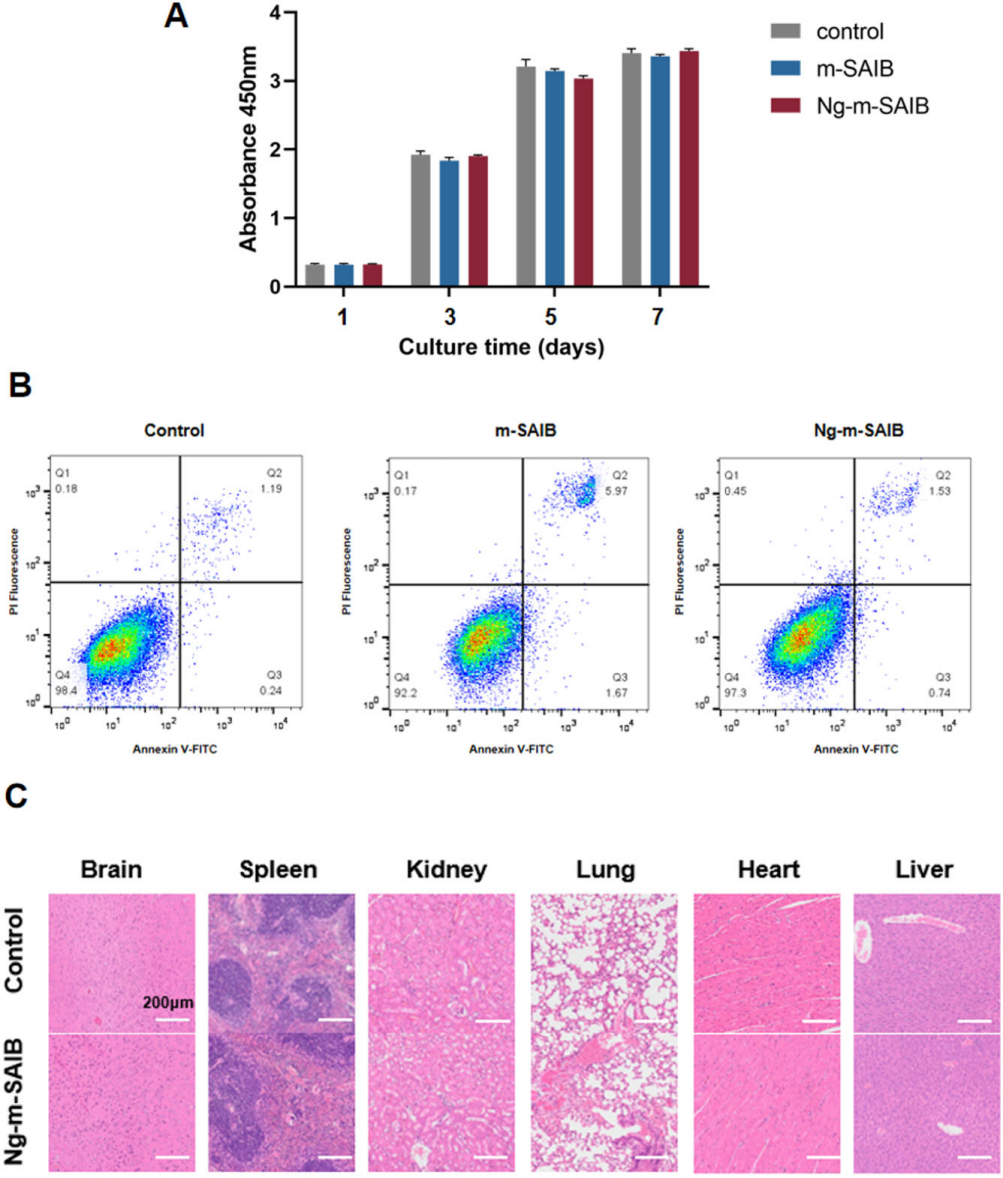
(**A**) Cell proliferation of RAW264.7 cultured with m-SAIB or Ng-m-SAIB was assessed using CCK8 assay. (**B**) Flow cytometric analysis of apoptosis. (**C**) H&E staining of the mice tissues in the control and Ng-m-SAIB groups after 8 weeks. Scale bar = 200 μm.

The CCK8 assay showed that the number of RAW264.7 cells in each group increased with time. Although the number of cells in the control group was relatively higher, there was no statistical difference in comparison to the other groups (Fig. 2A). The cytotoxicity test performed using flow cytometry showed consistent results as compared to those of the CCK8 assay (Fig. 2B). To examine the biocompatibility of the Ng-m-SAIB *in vivo*, the paraffinized sections of mice organs were stained using H&E to observe their histological morphologies. As shown in (Fig. 2C), the H&E staining results showed no significant changes in the brain, spleen, kidney, liver, lung and heart tissues in both groups.

### Polarization effects of Ng-m-SAIB on macrophages

**Figure 3.**
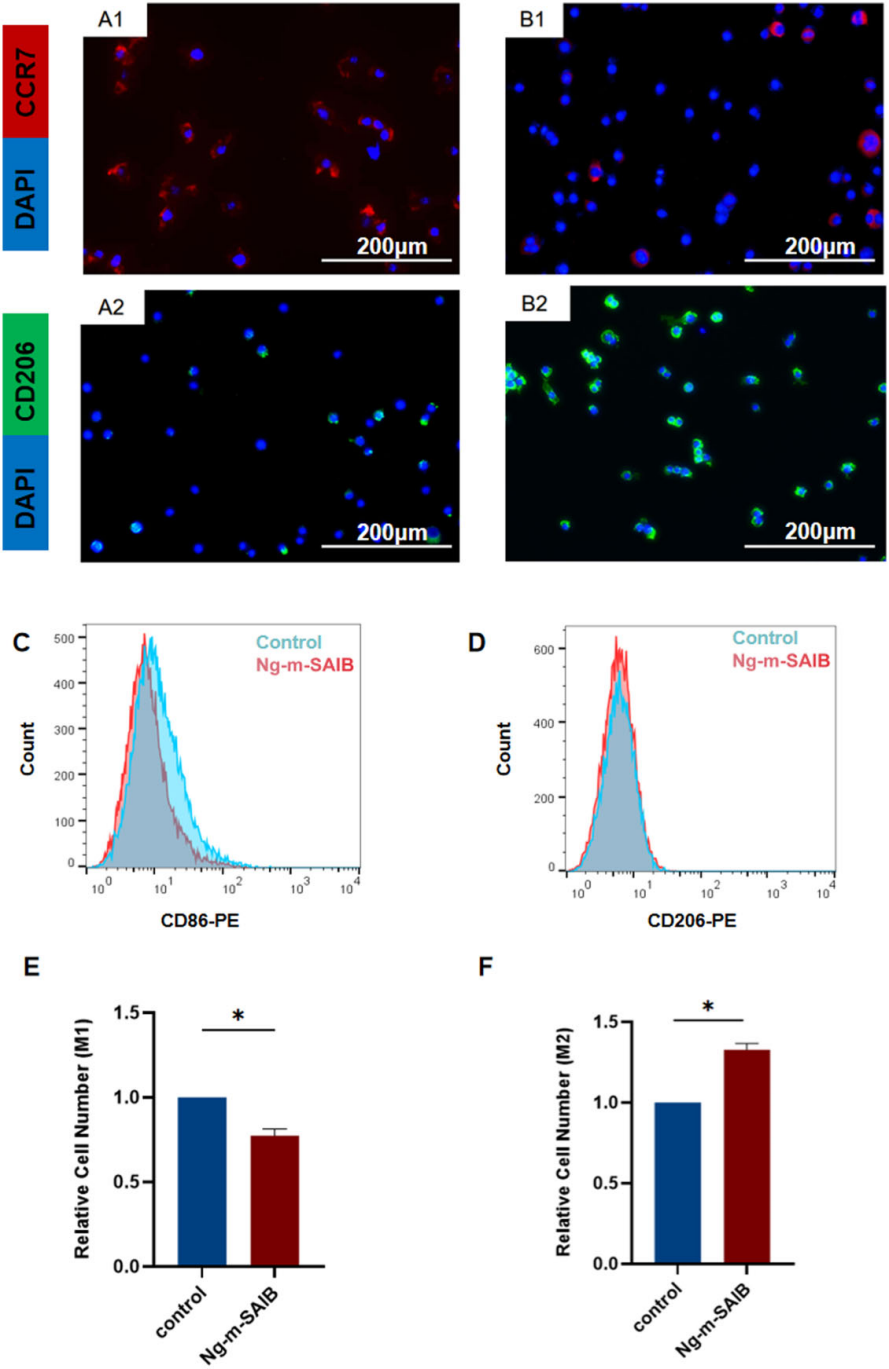
(**A1** and **A2**) Immunofluorescence staining results of RAW264.7 cells cultured without Ng-m-SAIB for 4 days. (**B1** and **B2**) Immunofluorescence staining results of RAW264.7 cells co-cultured with Ng-m-SAIB for 4 days. Scale bar = 200 μm. (**C**–**F**) Flow cytometry results of RAW264.7 cells co-cultured with or without Ng-m-SAIB for 4 days.

**Figure 4.**
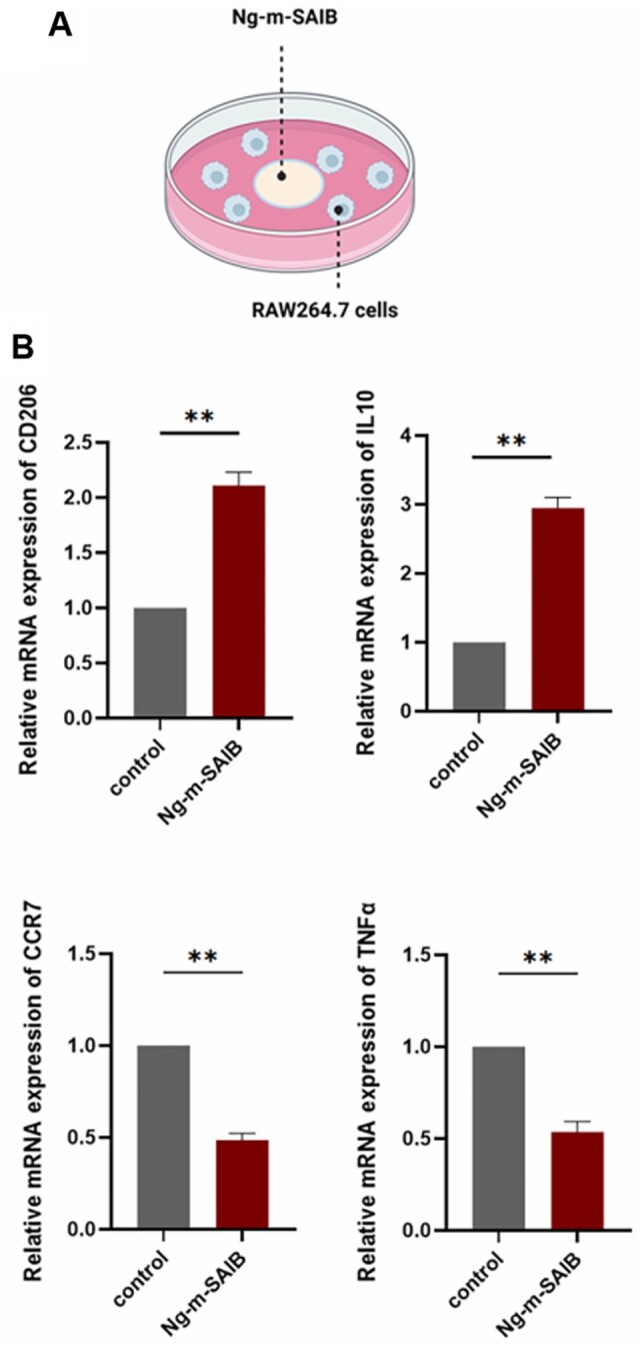
(**A**) Schematic diagram of RAW264.7 cells co-culture with Ng-m-SAIB. (**B**) Expression levels of *CD206*, *IL10*, *CCR7* and *TNFα* in the RAW264.7 cells co-cultured with or without Ng-m-SAIB for 4 days.

The immunofluorescence staining results of RAW264.7 cells (Fig. 3A1–B2) and the flow cytometry results (Fig. 3C and D) *in vitro* were consistent. The proportion of M2 macrophages in the Ng-m-SAIB group was higher than that in the control group, and the proportion of M1 macrophages was lower than that in the control group (Fig. 3E and F). The RT-PCR results also showed that Ng-m-SAIB significantly upregulated the expression of the *CD206* and *IL10* and decreased the expression of the *CCR7* and *TNFα* (Fig. 4B).

**Figure 5.**
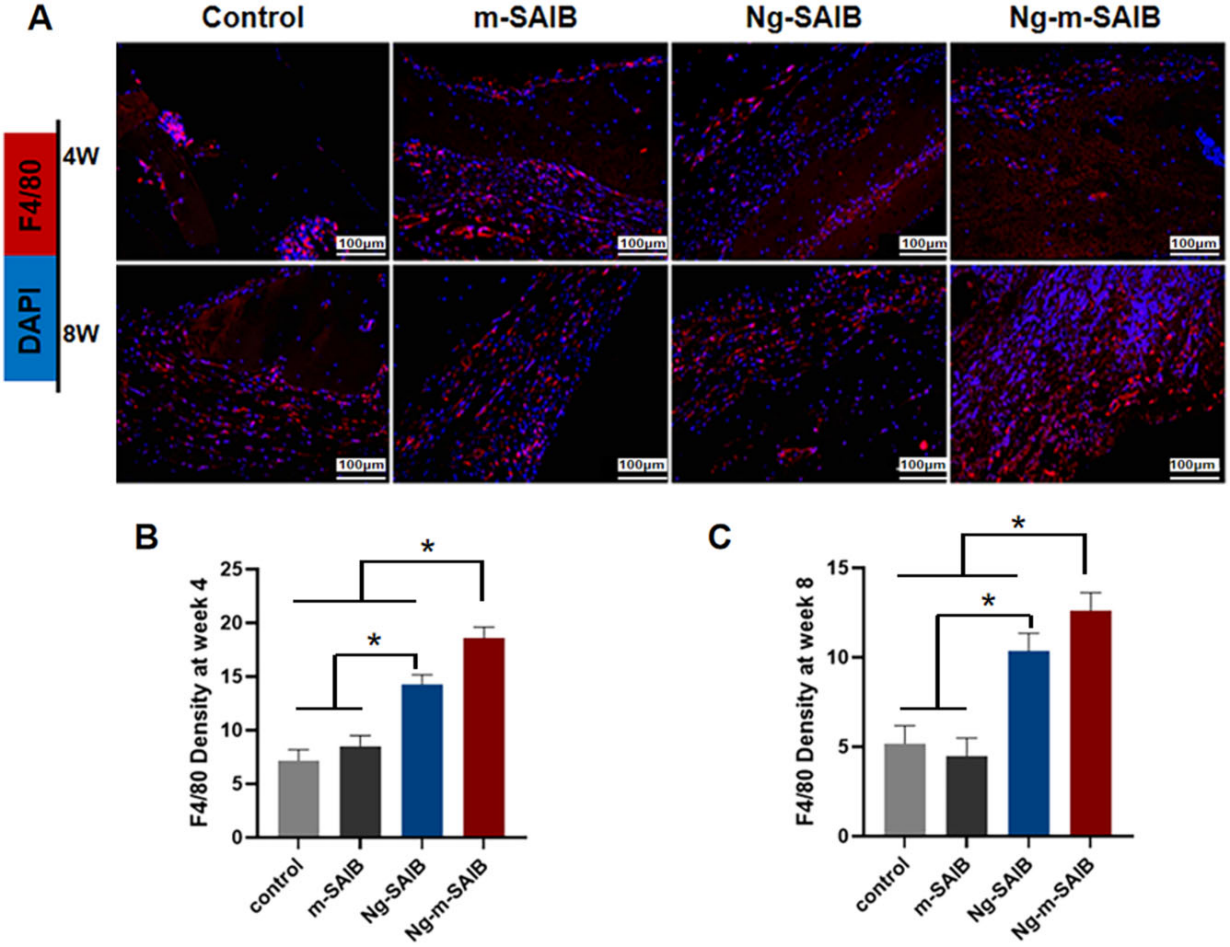
(**A**) Immunofluorescence staining results of *F4/80* (red) at the bone defects in cranial specimens (scale bar = 100 μm). Quantification of immunofluorescence intensity of F4/80 after (**B**) 4 weeks and (**C**) 8 weeks.

**Figure 6.**
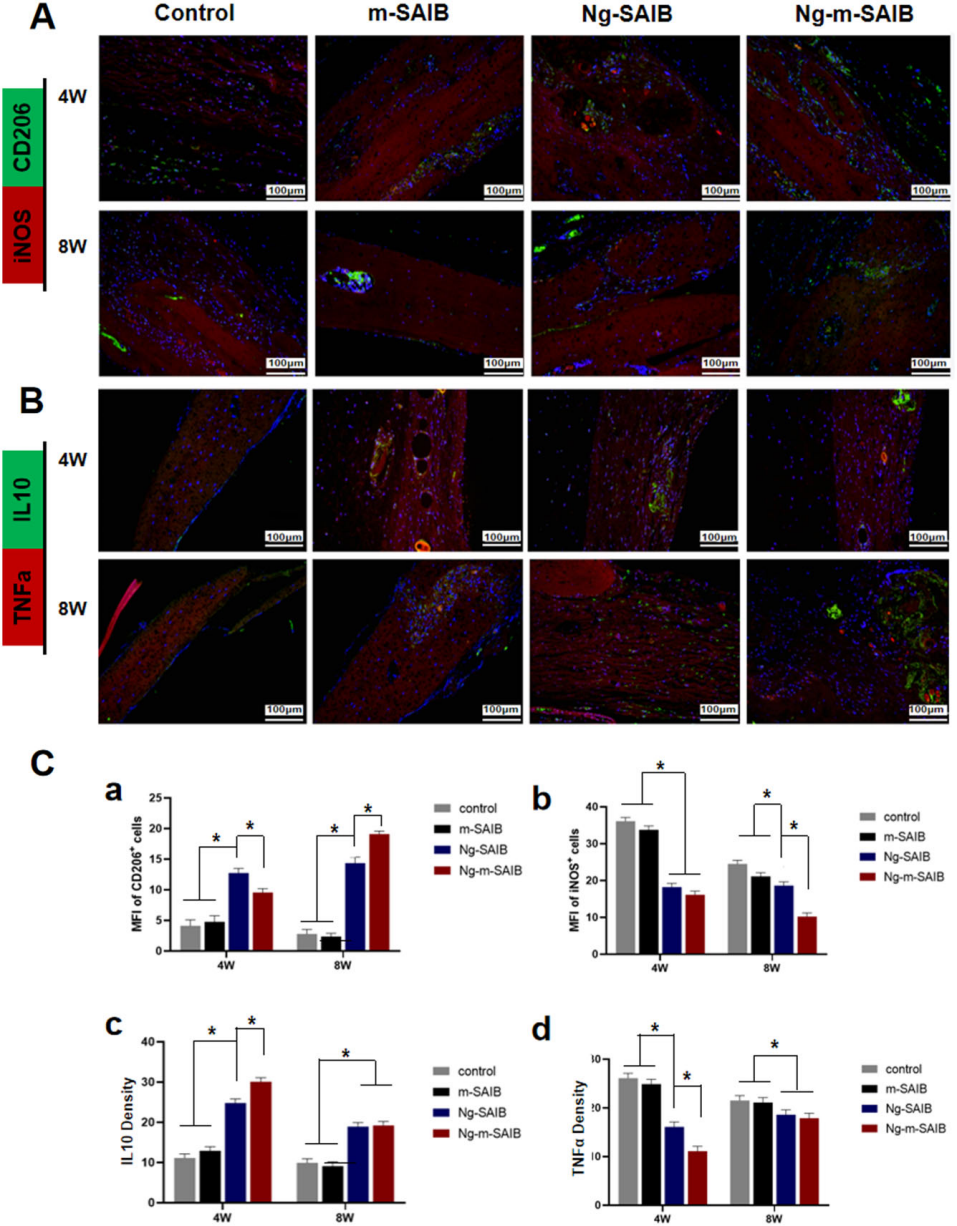
Immunofluorescence staining results of (**A**) *CD206* and *iNOS* and (**B**) *IL10* and *TNFα* at the bone defects in cranial specimens. Scale bar = 100 μm. (**C**) Mean fluorescence intensity (MFI) of *CD206*^+^ cells (a) and *iNOS*^+^ cells (b). Quantification of immunofluorescence intensity of *IL10* (c) and *TNFα* (d).

The results of *in vivo* experiments, showing the effects of Ng-m-SAIB on the polarization of macrophages, are shown in Figs. 5 and 6. The immunofluorescence results (Fig. 5) showed that all the groups had fewer *F4/80*-positive cells around the bone defects after 8 weeks of surgery as compared to those observed after 4 weeks of surgery; however, the Ng-m-SAIB group had consistently more *F4/80*-positive cells. As shown in Fig. 6A, after 4 weeks, the Ng-SAIB group had more *CD206*-positive cells than the Ng-m-SAIB group, and both these groups had higher *CD206*-positive cells than those in the control and m-SAIB groups. However, after 8 weeks, the Ng-m-SAIB group had the highest number of *CD206*-positive cells. *iNOS*-positive cells in Ng-m-SAIB group were the lowest at two time points. As shown in Fig. 6B and C, the highest amount of anti-inflammatory factor *IL10* and the lowest amount of pro-inflammatory factor *TNFα* were observed around the defect in the Ng-m-SAIB group at postoperative Week 4. At postoperative Week 8, no significant differences were observed between the Ng-m-SAIB and Ng-SAIB groups for *IL10* and *TNFα*, but both had more than the control group.

### Osteogenic differentiation effects of the conditioned medium

**Figure 7.**
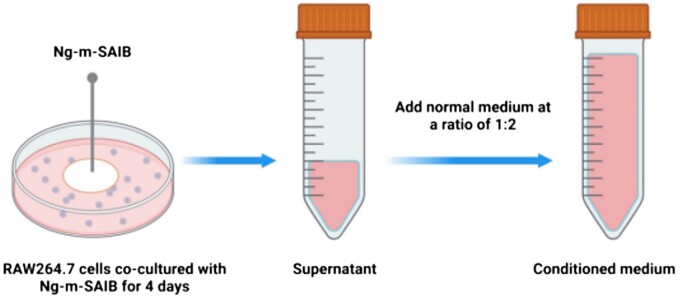
Schematic diagram of the preparation of conditioned medium.

**Figure 8.**
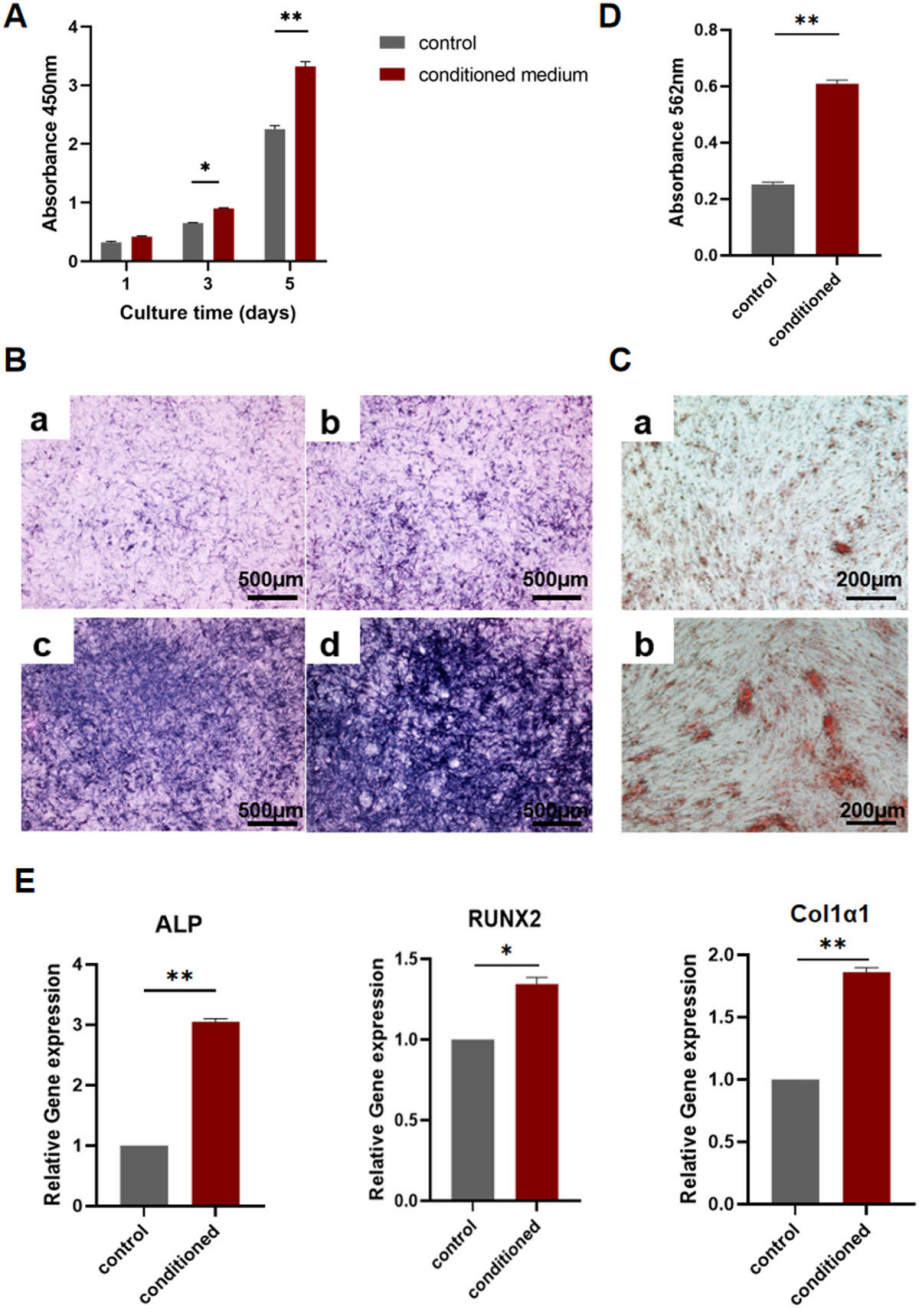
(**A**) CCK8 assay results after culturing BMSCs in different media. (**B**) (a and c) ALP staining results of BMSCs after incubation in normal medium for 7 and 14 days. (b and d) ALP staining results of BMSCs after incubation in the conditioned medium for 7 and 14 days. (**C**) Alizarin red staining results of BMSCs cultured in (a) normal medium and (b) conditioned medium for 21 days. (**D**) Semi-quantitative analysis of alizarin red staining. (**E**) Expression levels of *ALP*, *RUNX2* and *Col1α1* after 14 days of BMSCs incubation in different media.

The schematic diagram of the preparation of the conditioned medium is shown in Fig. 7. As shown in Fig. 8A, the number of BMSCs increased over time in both groups; however, the number of cells in the conditioned medium group was higher on both Days 3 and 5. And the ALP staining results also showed a higher expression level in the conditioned medium group (Fig. 8B). The alizarin red staining results are shown in Fig. 8D, and the absorbance results after calcium nodule lysis showed that more calcium nodules formed in the conditioned medium group (Fig. 8C). The RT-PCR analysis of osteoblast-related gene expression levels in each group showed a significant upregulation in the expression levels of three genes in the conditioned medium group (Fig. 8E).

### Osteogenic effects of Ng-m-SAIB on osteoporotic bone defects *in vivo*

**Figure 9.**
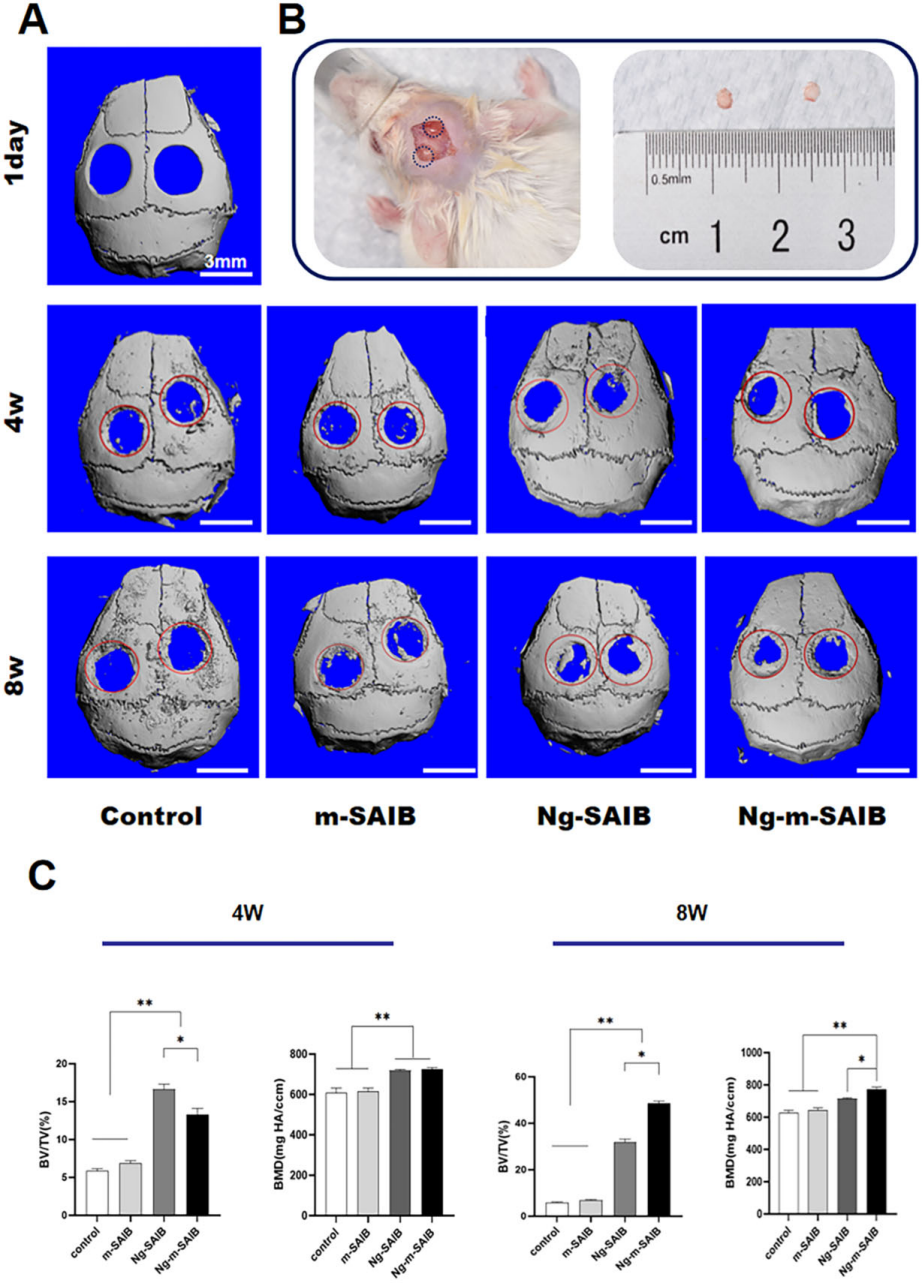
(**A**) Micro-CT images of the control, m-SAIB, Ng-SAIB and Ng-m-SAIB groups after 3D reconstruction. The scale bar was 3 mm. (**B**) Establishment of a SAMP6 mouse cranial critical defect model. The diameter was 3 mm. (**C**) BV/TV ratio of the defect and BMD changes during bone regeneration.

The 3D reconstructed cranial images are shown in Fig. 9A. After 4 and 8 weeks of surgery, both the control and m-SAIB groups showed very little new bone formation at the edges of the defect, showing no statistical difference between the two groups in the analysis of BV/TV (%) (Fig. 9C). The amount of new bone formation in both the Ng-SAIB and Ng-m-SAIB groups at both time points was greater than that in the control group and increased with the increase in time. After 4 weeks, the new bone formation in the Ng-SAIB group was greater than those in the Ng-m-SAIB; however, after 8 weeks, the Ng-m-SAIB group had the highest amount of new bone formation of all groups. Moreover, the mean BMD values of the Ng-SAIB and Ng-m-SAIB groups were significantly higher at both time points (Fig. 9C).

**Figure 10.**
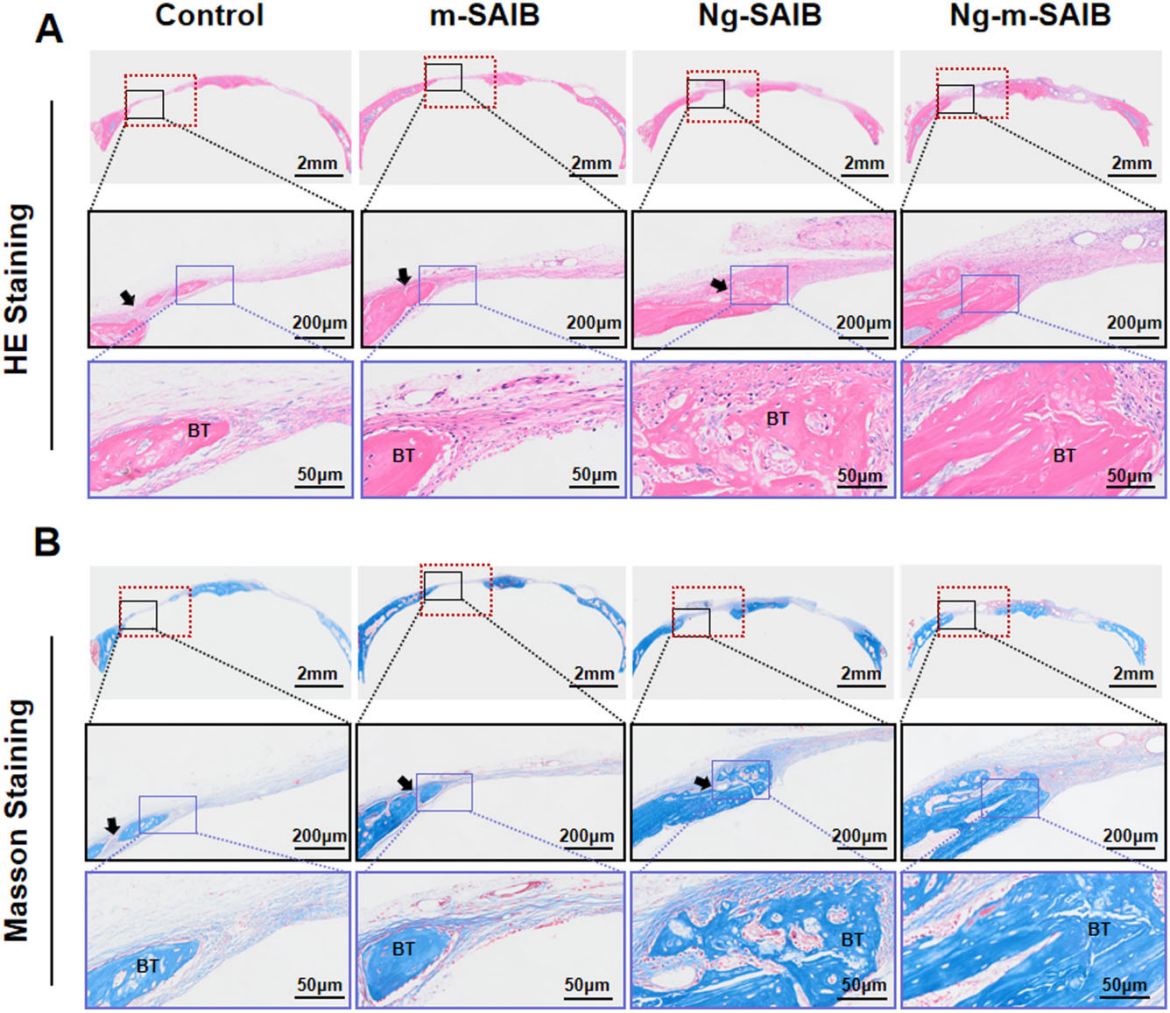
(**A**) H&E staining results of skull defect after 8 weeks of surgery. (**B**) Masson staining results of skull defect after 8 weeks of surgery. (The dotted line frame: scope of skull defect, diameter was 3 mm. The solid line frame: the enlarged image. The black arrow indicates the connective tissue at the edge of the new bone. BT = bone tissue.)

The results of the H&E and Masson staining (Fig. 10) showed that after 8 weeks of surgery, there was no significant inflammatory reaction in any group; however, there were bubble-like structures in the SAIB-containing group, which were incompletely degraded SAIB. Furthermore, 8 weeks after surgery revealed bone healing consistent with that observed by micro-CT. Although the bone defect remained open, newly formed bone was observed at the edges of the bone defects and had bone morphology similar to that of the original peripheral bone. Connective tissue was visible at the edges of some new bones in control, m-SAIB and Ng-SAIB groups.

**Figure 11.**
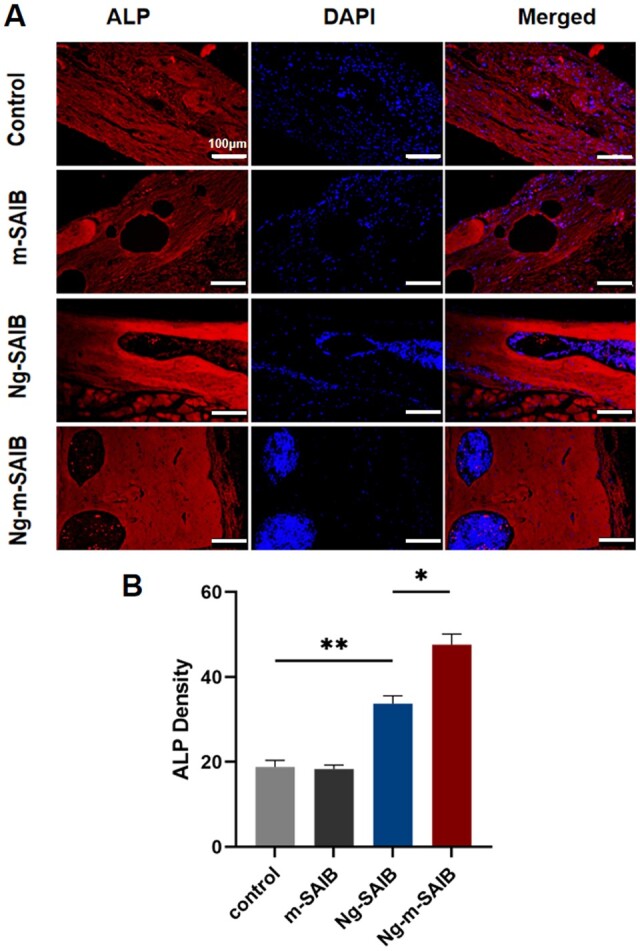
(**A**) ALP fluorescence staining results of skull defect after 8 weeks of surgery. Scale bar was 100 µm. (**B**) The result of average fluorescence intensity of ALP staining in each group.

The ALP immunofluorescence staining results are shown in Fig. 11A. The average fluorescence intensity analysis was performed on the fluorescent color pictures, as shown in Fig. 11B, the Ng-m-SAIB group had the highest average fluorescence intensity.

## Discussion

### Ng-m-SAIB could promote BMSCs’ differentiation via M2 macrophages

Bone tissue regeneration is a multi-system collaborative process; the immune system is an important target of bone-repairing materials while promoting bone formation [22, 23, 28]. When the biomaterials are implanted into the body, they activate the host’s innate immune system, and the innate immune cell subsets are the potential targets of biomaterials-mediated bone formation [35, 36]. Among the various natural immune cells, macrophages are the most important effector in biomaterial-related immune responses [37, 38]. Macrophages are divided into two major phenotypes, including M1 and M2 macrophages. Traditionally, the M1 macrophages are considered ‘pro-inflammatory’ cells, which exacerbate inflammation by producing cytokines such as *iNOS* and *TNFα*, thereby hindering tissue healing. In contrast, the M2 macrophages can produce the anti-inflammatory factor, such as *IL10*, to inhibit the progression of inflammation, thereby promoting tissue healing and repair [26, 39, 40]. Therefore, modulating the polarization of macrophages toward M2 might be an effective strategy for designing biomaterials to promote osteogenesis [41–43].

In the current study, Ng-m-SAIB was prepared. Both the *in vitro* and *in vivo* analyses demonstrated good biocompatibility. Ng-m-SAIB was co-cultured with RAW264.7 cells having direct contact. The flow cytometry and immunofluorescence staining results demonstrated that Ng-m-SAIB significantly promoted macrophage polarization toward M2, and the M2/M1 ratio was increased considerably. M2 macrophages enhance the production of the anti-inflammatory cytokine *IL-10*, which can promote the proliferation of chondrogenic cells, thereby facilitating osteogenesis [33, 44, 45]. Therefore, this study also identified the expression levels of *IL10* (anti-inflammatory) and *TNFα* (pro-inflammatory) secreted by macrophages under the action of Ng-m-SAIB using RT-PCR analysis. The results showed that Ng-m-SAIB could specifically enhance the expression level of IL10 and reduced that of TNFα *in vitro*. Moreover, the immunofluorescence staining results also showed that the Ng-m-SAIB group had the highest proportion of *F4/80*-positive cells in the cranial defects at both the time point (after 4 and 8 weeks) *in vivo*, and the relative levels of *CD206* and *IL10* were also the highest. These results indicated that Ng-m-SAIB could recruit more macrophages, promote the polarization of macrophages toward M2 and induce the secretion of anti-inflammatory factors.

To further verify the effects of cytokines secreted by Ng-m-SAIB-polarized macrophages on the osteogenic differentiation, the conditioned medium was prepared by directly co-culturing the Ng-m-SAIB with macrophages, and the BMSCs were incubated with the conditioned medium. The CCK8 assay results revealed that the conditioned medium promoted the proliferation of BMSCs, and the alizarin red and ALP staining results and RT-PCR results confirmed that the conditioned medium promoted osteogenic differentiation. In conclusion, these *in vitro* results demonstrated the indirect ‘immune osteogenic’ effects of Ng-m-SAIB.

However, there are still some things that could be improved in this experiment. Numerous factors might affect the polarization of macrophages. Besides the drug contained in the material, the particle size and shape of the material, pore size and porosity and material size and roughness might also affect the results [30, 33, 34, 45]. The present study only demonstrated the ability of Ng-m-SAIB to induce the M2 polarization of macrophages; however, the specific influencing factors were not investigated in detail. Subsequent experiments should be performed to further explore the effects of Ng-m-SAIB in depth in order to provide a theoretical basis to prepare Ng-m-SAIB with the best immune osteogenic effects.

### Osteogenic effects of Ng-m-SAIB on osteoporotic bone defects *in vivo*

Osteoporosis has been recognized as a progressive systemic disease. It is characterized by a reduction in bone mass and changes in the bone microstructure, which might likely cause fragility fractures [3]. The osteoblast-mediated bone formation in osteoporotic patients is significantly lower as compared to the osteoclast-mediated bone resorption; this leads to an osteogenesis/osteolysis imbalance in patients with osteoporosis. Therefore, the osteoporotic bone defects take longer to heal than normal bone defects, and in case of extensive bone defects, relaying on self-repair is more difficult [4, 46, 47].

Naringin has the dual effect of promoting osteogenesis and inhibiting osteolysis, so it can be used to treat osteoporosis [11]. Ng-m-SAIB possesses good long-term release properties of naringin as a sustained-release system [21], which can better perform the drug during long-term osteogenesis. Many studies have now reported the potential mechanism of naringin to promote osteogenesis [10, 48, 49]. Moreover, the results of the previous experiments of our group also demonstrated that Ng-m-SAIB had a good osteogenic effect on cranial defects in SD rats, which may promote osteogenesis by promoting the expression of *OCN* and *Runx-2* [20]. Studies also found that naringin could inhibit osteoclast formation and bone resorption by suppressing RANKL-induced activation of NF-kB and ERK [50]. Moreover, it could promote apoptosis of osteoclasts through the mitochondria-mediated apoptotic pathway, thereby inhibiting bone loss in the OVX rat model [10, 51]. In conclusion, Ng-m-SAIB was beneficial in promoting the osteogenic/osteolytic balance in osteoporotic bone defects.

However, naringin has not only direct action on bone cells as previously described. *In vitro* experiments in this study showed that Ng-m-SAIB could promote the polarization of macrophage toward M2 and secrete cytokines favorable to osteogenesis, achieving an indirect immune osteogenic effect. Therefore, we also conducted animal experiments to investigate its effect on promoting osteoporotic bone defects *in vivo*.

The SAMP6 mouse is an ideal animal model to study age-related osteoporosis. Numerous studies have confirmed that SAMP6 mice have essentially similar characteristics to those of human age-related osteoporosis [52–54]. Our previous study identified defects with a diameter of 2 mm or above as critical defects in SAMP6 mice [55]. Therefore, in the present study, a bone defect with a 3-mm diameter was made in the skull of SAMP6 mice, and Ng-m-SAIB was injected into the defected bone to observe its bone-forming ability. The micro-CT results showed that SAMP6 mice in the control group and the m-SAIB group formed a little new bone at the edge of the cranial defect with an average percentage of < 10% after 4 and 8 weeks of surgery. These results indicated poor autologous bone regeneration at the bone defect site in the osteoporotic state. In contrast, the Ng-SAIB and Ng-m-SAIB groups showed good bone formation effects, and the Ng-SAIB group (drug burst release group) showed more new bone formation at Week 4 as compared to the Ng-m-SAIB group. This might be because naringin in the Ng-SAIB group lacked the encapsulation and restriction of microspheres, thereby releasing more naringin in the early stages. However, at Week 8, the Ng-m-SAIB group had the highest amount of new bone formation. This might be related to the long-term effective release of naringin in the Ng-m-SAIB group [21]. From a pharmacological perspective, the drug delivery system applied to bone tissue engineering could reduce the toxic effects caused by the high dose of burst release and extend the duration of drug action at the point of administration, thereby benefiting osteogenesis [56, 57].

The ability of numerous biomaterials to promote osteogenesis in osteoporotic bone defects has been recently demonstrated. Although the alendronate-loaded scaffold developed by Zeng et al. promoted the repair of cranial defects in osteoporotic SD rats, the problem of burst drug release from this scaffold still requires further improvement [58]. Chu et al. constructed the lanthanum-substituted layered double hydroxide nanohybrid scaffolds and placed them in the critical skull defects of osteoporotic SD rats. After 12 weeks, the rats with this scaffold showed a significant formation of new bone [59]. It was difficult to evaluate the osteogenic effects of Ng-m-SAIB on osteoporotic bone defects using other biomaterials as positive controls due to differences in administration methods and other factors, such as the material implantation site and material degradation time, which might affect the final results. However, the *in vivo* results confirmed the osteogenesis-promoting effects of Ng-m-SAIB in the osteoporotic bone defects in comparison with the control, m-SAIB and Ng-SAIB groups.

In this study, the dose of naringin used in *in vivo* experiments was selected based on the results of *in vitro* experiments and the dosage used in previous literature [20, 60–62]. In order to achieve the ideal effects, naringin should be controlled within a certain dosage range. If the dose is too low, its effects of promoting osteogenesis and inhibiting osteoclasts might not obvious; if the concentration is too high, it might inhibit osteogenesis [10, 63]. Therefore, the optimal dosage for *in vivo* osteogenesis requires further investigation. The osteogenic observation period for *in vivo* experiments can be further clarified based on the results of subsequent *in vivo* release and degradation of Ng-m-SAIB.

## Conclusions

In summary, Ng-m-SAIB is a sustained drug delivery system having good biocompatibility. It can promote the polarization of macrophages toward M2, thereby forming a favorable immune microenvironment and achieving the ‘immune osteogenesis’ effect. Also, Ng-m-SAIB has ‘direct osteogenesis’ effects on the repair of osteoporotic bone defects. Although the specific mechanism of cytokines secreted by macrophages in osteogenesis remains to be further investigated, the experimental results of the present study still demonstrate that Ng-m-SAIB might be a promising biomaterial having the potential of promoting osteogenesis in osteoporotic bone defects.

## Supplementary Material

rbad006_Supplementary_Data
