# Supplementary material for: Electrospun naringin-loaded microsphere/sucrose acetate isobutyrate system promotes macrophage polarization toward M2 and facilitates osteoporotic bone defect repair
Source: Regen Biomater. 2023 Feb 20;10:rbad006. doi: 10.1093/rb/rbad006 (PMC9998078; doi:10.1093/rb/rbad006)
Supplement: rbad006_Supplementary_Data [file rbad006_supplementary_data.zip › Supplementary Material.docx]

**Supplementary Material**

1. **Preparation of Ng-m and Ng-m-SAIB**

Firstly, Poly(ethyleneglycol) (PEG), Poly(lactide-co-glycolide) (PLGA), and naringin (Ng) was added to the chloroform solution in a specific weight ratio (1:20:3:15000) and stirred magnetically for 2 hours. Then the fully dissolved solution was loaded in a 5 ml disposable syringe with a 22G stainless steel needle. Next, a single-nozzle electrospinning setup (Beijing Yongkang Leye Technology Development Co Ltd, China) was used to prepare microspheres (Figure 2). The parameters used in the experiment are as follows: constant flow rate was 0.120 mm/min, applied voltage was 18 kV, and the collection distance of the microspheres was 15 cm. The ambient temperature range for the preparation of microspheres was 22 ℃ to 23℃, and the humidity range was 30% to 31% [1]. The microspheres collected on tin foil were dried in a constant temperature oven at 37℃ for 48 hours, and then the dried microspheres were collected into clean EP tubes and stored in a -20℃ refrigerator away from light. The required amount of naringin-loaded microspheres was added to SAIB (Sigma-Aldrich, US), and Ng-m-SAIB were obtained by magnetic stirring for 5 min.

1. **Drug release and degradation of Ng-m and Ng-m-SAIB**

As shown in Table 1, the drug encapsulation efficacy (EE) and drug loading efficiency (LE) of Ng-m were 89.01% and 11.48%, respectively, and the contact angle was about 49.51°.

As shown in Figure 2A and Figure 2B, Ng-m-SAIB significantly improved the drug burst release phenomenon of Ng-m and was able to release naringin for an extended period. The experimental results showed that its drug release behavior lasted until the 92nd day; this may be related to the rapid change in the porosity of Ng-m-SAIB, where a larger porosity represents an easier exchange of the drug with the external aqueous phase, resulting in a faster drug release rate [2, 3]. As shown in Figure 2D, the porosity of Ng-m-SAIB changed faster until 15 days ago and then stabilized.

| **Ng-m** | **Diameter,um** | | **CV,%** | | **EE,%** | **LE,%** | | **Contact Angle** | | **Water Absorption,%** | |
| --- | --- | --- | --- | --- | --- | --- | --- | --- | --- | --- | --- |
|  | **5.03±0.172** | **3.41±0.003** | | **89.01±0.15** | | | **11.48±0.07** | | **49.51±1.25** | | **97.20±8.17** |

**Table 1.** The characters of Ng-m.


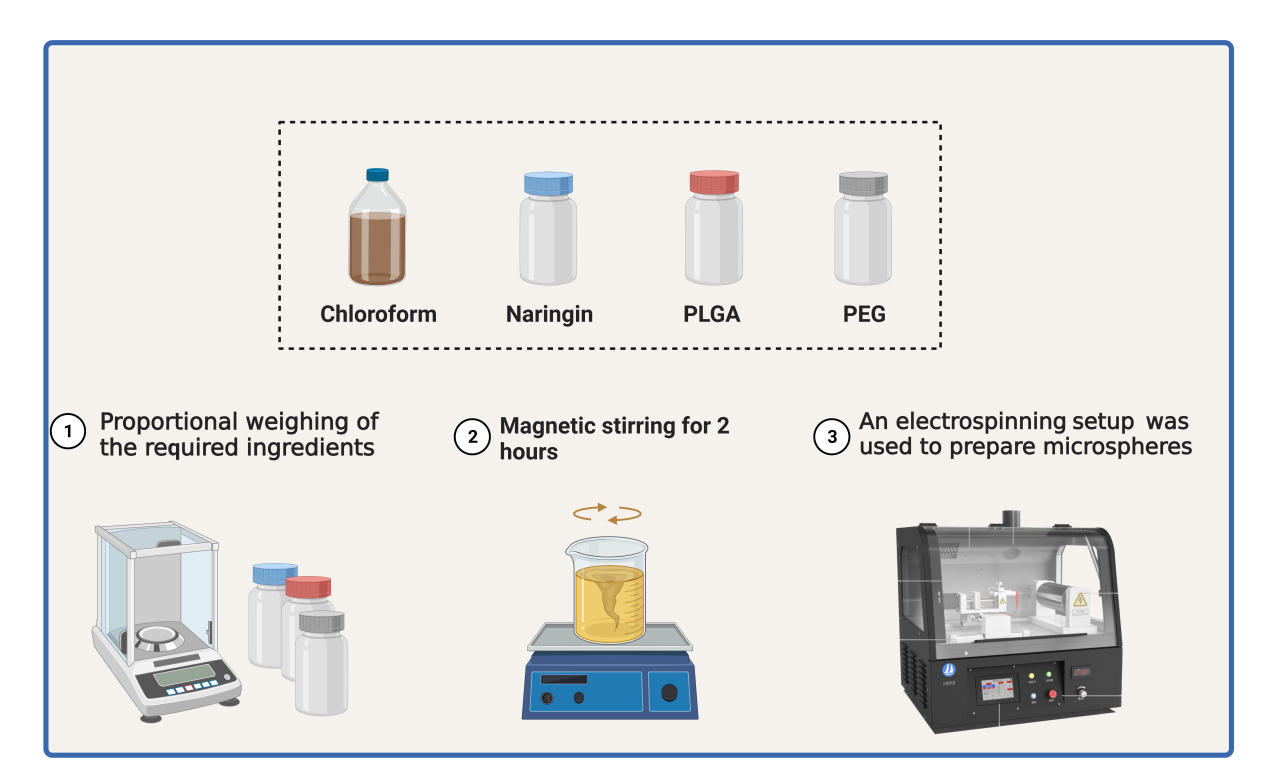
**Figure 1.** Schematic diagram of the fabrication of naringin-loaded microspheres.


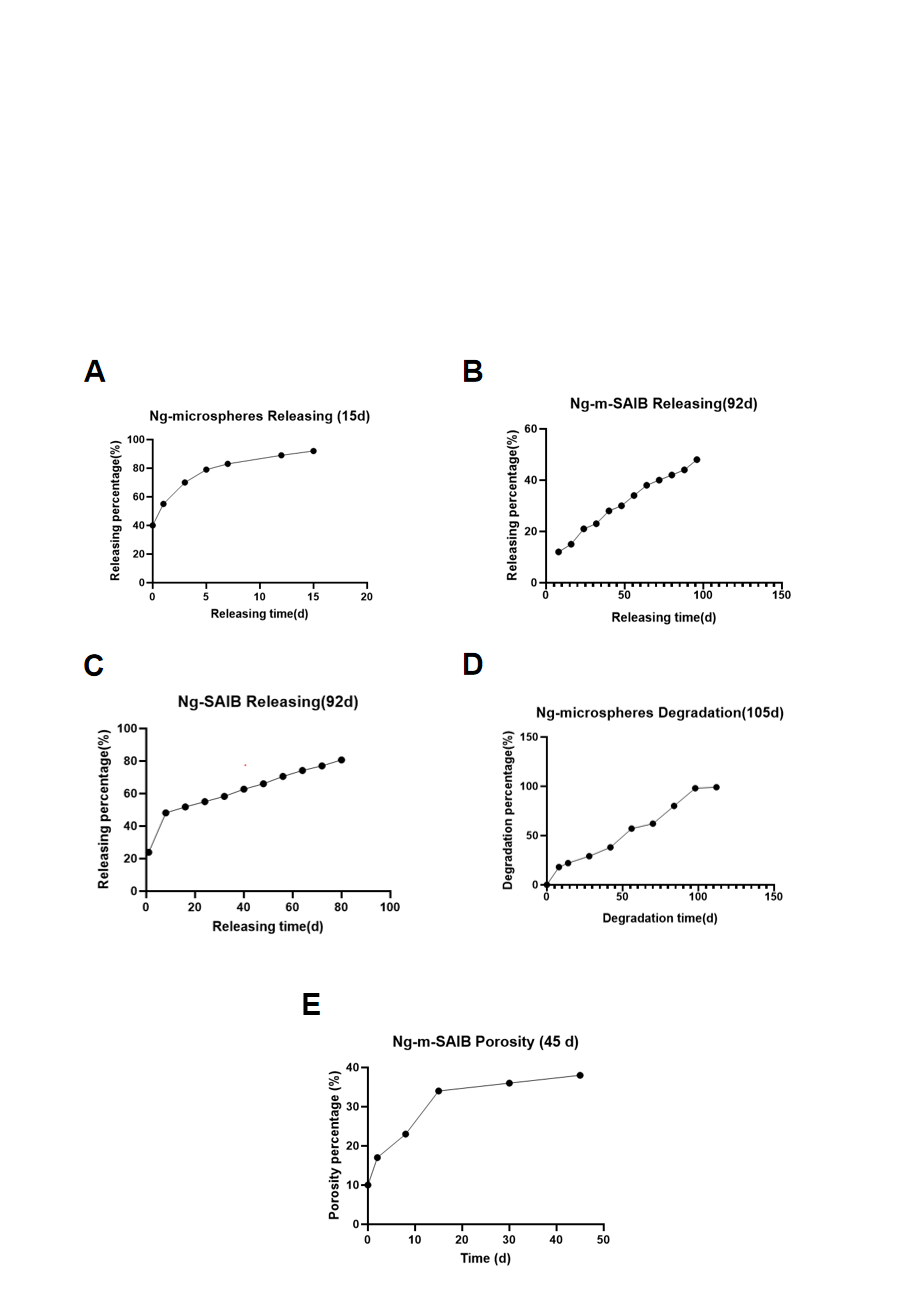


**Figure 2.** (A) In vitro drug release of Ng-m. (B) In vitro drug release of Ng-m-SAIB. (C) In vitro drug release of Ng-SAIB. (D) Degradation of Ng-m in vitro. (E) In vitro porosity of Ng-m-SAIB.

**Reference**

1. Zhang D, Song J, Almassri HNS, Ma Y, Zhang T, Cheng Y, Wu X. Effect of microsphere size on the drug release and experimental characterization of an electrospun naringin‐loaded microsphere/sucrose acetate isobutyrate (SAIB) depot. *Polymers for Advanced Technologies* 2020;31:1110-1121.
2. Lin X, Yang S, Gou J, Zhao M, Zhang Y, Qi N, He H, Cai C, Tang X, Guo P. A novel risperidone-loaded SAIB–PLGA mixture matrix depot with a reduced burst release: effects of solvents and PLGA on drug release behaviors in vitro/in vivo. *Journal of Materials Science: Materials in Medicine* 2011;23:443-455.
3. Shamma RN, Elkasabgy NA, Mahmoud AA, Gawdat SI, Kataia MM, Abdel Hamid MA. Design of novel injectable in-situ forming scaffolds for non-surgical treatment of periapical lesions: In-vitro and in-vivo evaluation. *Int J Pharm* 2017;521:306-317
