# Supplementary material for: Electrospun naringin-loaded microsphere/sucrose acetate isobutyrate system promotes macrophage polarization toward M2 and facilitates osteoporotic bone defect repair
Source: Regen Biomater. 2023 Feb 20;10:rbad006. doi: 10.1093/rb/rbad006 (PMC9998078; doi:10.1093/rb/rbad006)
Supplement: rbad006_Supplementary_Data [file rbad006_supplementary_data.zip › reponce letter.docx]

**21-Jan-2023**

**Response Letter to *Regenerative Biomaterials***

**Paper ID:**  RB-2022-258

**Paper Title:** Electrospun naringin-loaded microsphere/sucrose acetate isobutyrate (SAIB) system promotes macrophage polarization toward M2 and facilitates osteoporotic bone defect repair

Dear editor and all reviewers,

We gratefully thank the editor and all reviewers for their time spent making constructive remarks and valuable suggestions, which have significantly raised the quality of the manuscript. We have considered all these comments and suggestions and made corrections in the revised manuscript, and all changes were marked in blue. Our point-by-point responses are detailed below.

**Review Editor**

**Comments:**

The graphic abstract could be re-designed owning to the texts in the present GA are too small to be legible.

**Reply:**

Thank you for your important reminder. We have re-designed the GA.

[Origin]:

[Revised]

**Reviewer : 1**

**Comments:**

I have raised the issue of histological section results in previous reviews. Although the author re-selected the more effective staining images and modified the corresponding descriptions, there are still some issues in this section. Page 57 line 53-57, according to the author’s description, “New bone formation was seen around the skull defects in all four groups of mice, with more new bone matrix in the Ng-m-SAIB and Ng-SAIB groups.” However, the evidence of the histological section representing bone tissue was still not observed in the Ng-SAIB group (Figure 10). Furthermore, H&E and Masson staining results of skull defect after 4 weeks of surgery should be provided. Please add the scale bars of the enlarged figures.

**Reply:**

Thank you so much for your careful check. We apologize for not showing satisfactory staining results in the previous revision. We have carefully examined the staining images and re-selected two enlarged areas in each group of specimens to better reflect the differences in osteogenesis among the groups and added the appropriate scale (Page 34).

Because the mice used in our animal experiments were osteoporotic model mice with poorer osteogenesis than ordinary mice, and the postoperative CT scans showed little new bone in the fourth week, we only selected specimens from the eighth week postoperatively for HE and Masson staining. We apologize for the error in describing the staining results in the first draft regarding the "fourth postoperative week" and have corrected it in the second draft.

Thank you again for your valuable comments, which have enabled us to continuously improve the quality of our manuscripts. We will apply the valuable experience of this revision in writing subsequent articles.

[Origin]:

The results of the H&E and Masson staining (Figure 10) showed that after 8 weeks of surgery there was no significant inflammatory reaction in any group; however, there were bubble-like structures in the SAIB-containing group, which were incompletely degraded SAIB. New bone formation was seen around the skull defects in all four groups of mice, with more new bone matrix in the Ng-m-SAIB and Ng-SAIB groups, and the bone defects in the Ng-m-SAIB group showed the most uniform red and blue staining regions.

**Figure 10.** (A) H&E staining results of skull defect after 8 weeks of surgery. (B) Masson staining results of skull defect after 8 weeks of surgery. Scale bar = 2 mm. The red dotted line frame and black solid line frame show the defect and enlarged image, respectively.

[Revised]

The results of the H&E and Masson staining (Figure 10) showed that after eight weeks of surgery, there was no significant inflammatory reaction in any group; however, there were bubble-like structures in the SAIB-containing group, which were incompletely degraded SAIB. Furthermore, eight weeks after surgery revealed bone healing consistent with that observed by micro-CT. Although the bone defect remained open, newly formed bone was observed at the edges of the bone defects and had bone morphology similar to that of the peripheral original bone. Connective tissue was visible at the edges of some new bones in control, m-SAIB, and Ng-SAIB groups.

**Figure 10.** (A) H&E staining results of skull defect after 8 weeks of surgery. (B) Masson staining results of skull defect after 8 weeks of surgery. (The red dotted line frame : scope of skull defect, diameter was 3 mm. The black and blue solid line frame: the enlarged image. The black arrow indicates the connective tissue at the edge of the new bone. BT = bone tissue )

**Reviewer : 2**

**Comments:**

The author has addressed all my concerns. I recommend to accept this manuscript.

**Reply:**

Thank you very much for your recommendation! Your previous valuable comments have allowed us to improve the quality of our manuscripts.
